# Patients’ perception of changes and consequences after tumor resection: A qualitative study in Austrian patients with musculoskeletal malignancies

**DOI:** 10.1007/s00508-022-02136-6

**Published:** 2023-01-03

**Authors:** Carmen Trost, Stephan Heisinger, Philipp T. Funovics, Reinhard Windhager, Gerhard M. Hobusch, Tanja Stamm

**Affiliations:** 1 Department of Orthopedics and Trauma Surgery, Medical University of Vienna, Waehringer Guertel 18–20, 1090 Vienna, Austria; 2 Institute for Outcomes Research, Medical University of Vienna, Vienna, Austria

**Keywords:** Qualitative research, Health-professional-patient communication, Patients’ perception, General practitioners, Musculoskeletal malignancies, Patients’ needs

## Abstract

**Objective:**

The aim of this study was to investigate the effects and consequences of surgical treatment of patients with musculoskeletal malignancies on everyday life.

**Methods:**

A modified form of grounded theory was used for data collection and analysis. Data collection was systematic and analyzed simultaneously and 16 interviews were conducted: 2 narrative, 11 guided and 3 expert interviews (surgeon, physical therapist, support group). Data collection and analysis alternated until no new codes could be found. Once theoretical saturation was achieved, the main category was formed and described using the literature.

**Results:**

The main category results from the combination of all categories and leads to the core category. In the center is the affected person and in the immediate environment are the patient’s relatives/partners. In the next instance the primary care physician is necessary to establish a sense of normalcy. This depends on the individuality of the person and the restored possibilities of movement.

**Conclusion:**

Based on the results, the necessity of implementing psychosocial care involving the social environment is shown. The importance of relatives/partners for recovery is emphasized. Furthermore, the communication between the specialists and family physicians should be simplified.

**Supplementary Information:**

The online version of this article (10.1007/s00508-022-02136-6) contains supplementary material, which is available to authorized users.

## Introduction

The World Health Organization (WHO) stated that 71% of deaths are due to noncommunicable diseases; cancer is the second most common of the four most noncommunicable diseases [1]. The diagnosis of sarcoma and other musculoskeletal malignancies and the associated treatment, including surgery, is a life-changing and difficult process for patients [2–5].

The research for musculoskeletal malignancies has increased since the early 1960s, in particular regarding treatment and medical therapy. In the literature it could be shown that patients diagnosed with osteosarcoma of the lower extremity experienced reduced mobility and other negative impacts on work/education after treatment [6]. Getting back to normality depends on the personality and physical activity of the affected person [3, 7]. The perception of the disease in the social environment and in the daily life of patients with malignant diseases of the musculoskeletal system has not yet been studied in Austria [8–11]. The use of qualitative methods provides important information about aspects of the patients’ illness that may be neglected in commonly used self-reported questionnaires. A questionnaire cannot query communication structures. Qualitative research forms the basis for understanding psychosocial facts and structures in order to be able to name the way to normality.

After the diagnosis of malignant disease of bone or soft tissues, patients are confronted not only with their diagnosis, but also with the need for extensive surgery, functional limitations and other comorbidities [12]. In order to counsel patients and their families, it is essential to collect and analyze patients’ respective experiences [13]. Questions about patients’ experiences and perceptions cannot be answered with standardized questionnaires because the individual can only choose between the given answer options. Qualitative research is needed to answer these questions [14–16]. The grounded theory method is the tool of choice for mapping phenomena embedded in social contexts [17].

The purpose of this study was to investigate how patients with sarcoma and other musculoskeletal malignancies recover to a daily routine after surgery/treatment.

## Methods

A qualitative interview study was conducted. following a modified form of Charma’s grounded theory (GT) This is a research method that develops a new theory or modifies an existing one based on data collected and analyzed systematically and simultaneously [15]. Analytic codes and categories are constructed from data, not from preconceived derived hypotheses [18]. In this continuously comparative method, comparisons are made at each stage of the analysis. It promotes theory development at each step of data collection and analysis. Memo writing is important for elaborating categories, specifying their properties, defining relationships between categories, and identifying gaps [18, 19]. The ethics committee of the local university approved and reviewed this study. All participant signed an informed consent form.

### Sampling and recruitment

The purposive sampling in this study aimed at diversity for rich theory building rather than population representativity. Patients treated at our hospital between February 2015 and March 2019 were asked to participate in this study after providing written informed consent. The inclusion criteria were musculoskeletal tumor surgery with (i) consecutive physical limitation or (ii) a regular treatment more than 6 months ago, such as: injections, chemotherapy, radiation, physical therapy. In addition, participants had to be at least 18 years old and were included regardless of gender and ethnicity. The exclusion criteria were (i) patients under 18 years old, (ii) insufficient knowledge of German.

In the study six semi-structured interviews (marked 1) were conducted to develop the initial theory. For development and consolidation, two narrative interviews were conducted: one with a female and one with a male of different age groups; then five adapted semi-structured (marked 2) and three expert interviews were conducted. This iterative process is intended to obtain a theoretical sampling showing that more information cannot be obtained with more questions [20].

Based on the available data and experiences, the questions for the semi-structured interviews were formulated. The guided interviews consisted of three parts: a general part in which respondents were asked questions such as: “Please tell me about your disease and treatment.” (1, 2). A second part provided information about the current status: “How much is your disease present in everyday life?” (1), “How are you today?” (2), “Would you tell me about your family/home?” (2), or “How do you explain your disease to someone else?” (2), “What is your understanding of your disease after surgery?” (1). In the third part, some sociodemographic questions were investigated: “How old are you?” (1, 2), “Are you employed?” (1, 2). Two interviews were designed as narrative interviews, and the basic structure of this interview was free narrative. The opening question was open ended: “Due to your illness you have had many treatments, surgeries, and examinations. Please tell me about your experience of this and what this experience has done to your outlook on life?” There are four phases of a narrative interview: (1) the introductory question, which should not guide or limit the interviewee. (2) It is important not to interrupt the person speaking, and the interviewer should only make supportive gestures and comments to maintain communication. (3) In the second phase, open-ended questions can be discussed and (4) the conclusion of the interview [21].

For the expert interviews, a surgeon from the local department who was not involved in the project was interviewed. Furthermore, a physiotherapist, also from the department. The chair of the support group GIST Support Austria made himself available for an expert interview [22]. The support group provides information and advice for patients and those affected. It also organizes meetings to exchange experiences. The topics of the group are: cancer and rare disease self-help. Out of necessity, since there is no general support group for musculoskeletal diseases in Austria, those affected find themselves in support groups for, e.g. GIST (soft tissue sarcomas) or War Victims and Disabled Persons Association (KOBV).

The interviews were audio-recorded, pseudonymized and transcribed [23], the data are stored password and access-protected.

### Data collection

All 16 interviews were conducted by the author CT to ensure consistency in the data collection process. The patient interviews were conducted during waiting times, so no separate appointment was necessary. The expert interviews (including support group) were also conducted in outpatient rooms at our hospital.

### Data analysis

Data analysis was conducted using the GT method by the author CT in collaboration with students from peer groups and members of our and another group. Feedback was provided during the course or meetings with other students. The analysis was performed using Atlas.ti.

### Coding

**Fig. 1:**
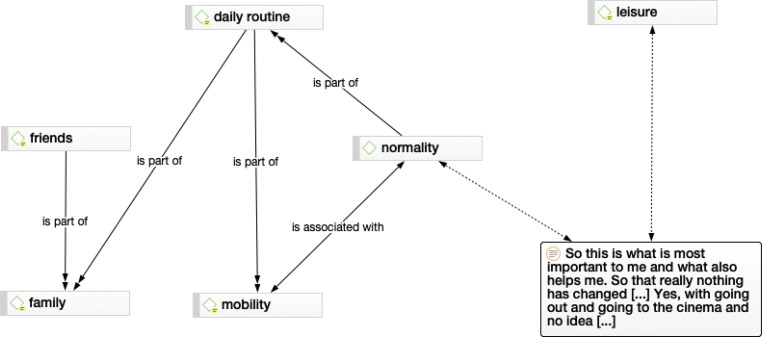
Normality network. Normality is part of the daily routine, which is part of mobility and family. The normality is related to the mobility

**Fig. 2:**
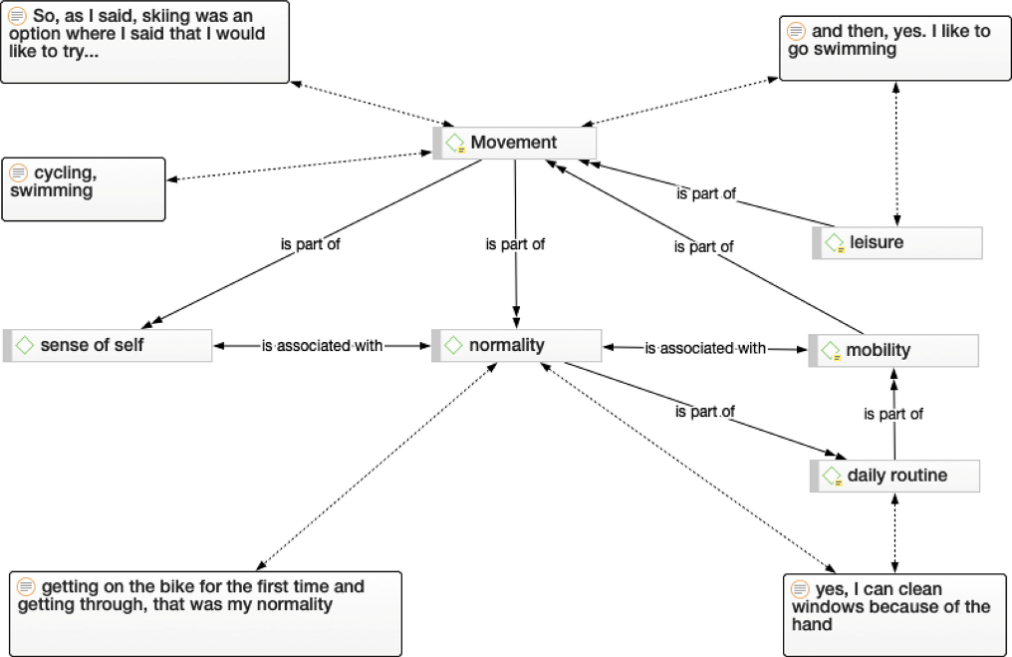
Movement network. Connections and nodes visualize how movement is cross-linked in the daily life. This figure shows which activities the interviewee mentioned for example: swimming, cycling, skiing and cleaning the windows

**Fig. 3:**
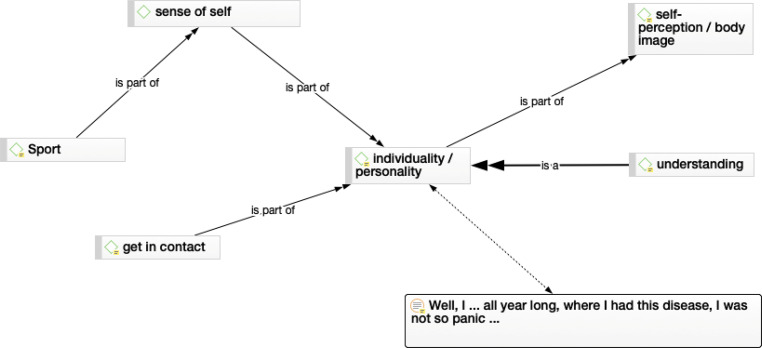
Individuality/personality network. The personality from the affected person explains their acting, for instance: getting in contact with other patients, or their understanding of their own situation

**Fig. 4:**
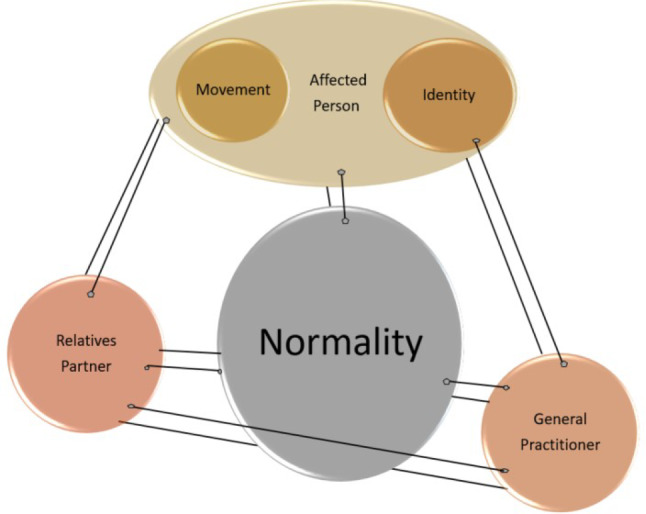
This picture shows the theoretical model of the affected person to find into a way of normality, influenced by the level of movement and the identity of the patient. For reaching the normality status the support of the relatives/partners and general practitioner is needed

Open, axial and selective coding were applied in this iterative cyclic process: The first step was to analyze line by line and create in vivo codes (open coding). In vivo codes are descriptions alongside the text; the code is based on the participant’s statement. For example, diagnosis: each interviewee talked about when they received the diagnosis. This occurred at the beginning of the interview situation and they began to narrate. Then, the in vivo codes were reread and summarized into codes and concepts. In the case of the diagnostic interview, it was conceptualized as doctor-patient communication and later as part of the support provided by the general practitioner (GP) [18, 19]. Axial coding was used to relate the concepts: “contradicts”, “is a”, “is associated with”, “is a cause of”, “is part of”, “is property of”. The relationships (relatives/partners and GP) are connected to the phenomenon focused on the category or referred to as the network formation process (see Figs. 1, 2 and 3). The threads converge across these axis categories to form a core that represents the core category (selective coding). The resulting network then gives rise to the theory about the phenomenon under study (Fig. 4). Memo writing is an essential and continuous process to develop the theory. Each decision or observation was noted. The structures of the codes were analyzed and visualized.

### Data saturation

Data saturation was reached after no new codes could be found. From the eighth interview on, no new concepts could be found within the codes. At this point, the quotes and categories filled the concepts and no new findings will change these results. Again, this is an iterative cyclical process with discussions and visualization, for instance mind mapping, colored sheets, writing memos and reading literature [24, 25].

## Results

### Participant characteristics

Table 1 shows an overview of the descriptive data of the participants. The final sample consisted of six semi-structured interviews (1) with total time 02:59 h and 0:29 h mean, two narrative interviews with a total time of 1:16 h and an average of 38 min, and five guided interviews (2) with total time 3:24 h and 0:41 h average. Overall, seven females and six males participated, with median ages of 50.1 and 50.7 years, respectively. In respect of the anonymity of the experts the information of their age will be not shown. The interview time of the experts was in summary 2:08 h and 56 min average. Summarized the interview time was 09:47 h. The second table lists the diagnoses and treatments of the participants (Table 2).

**Table 1:** Descriptive table of the interview time, age, and gender of the participants

IP	Duration hh:mm:ss	Age (years)	Female (*n* = 8)	Male (*n* = 8)	Narrative (*n* = 2)	Duration guided 1 (*n* = 6)	Duration guided 2 (*n* = 5)	Duration expert (*n* = 3)
Guided 1	00:46:59	25	25	–	–	00:46:59	–	–
Guided 1	00:53:07	50	50	–	–	00:53:07	–	–
Guided 1	00:14:10	46	–	46	–	00:14:10	–	–
Guided 1	00:22:03	66	–	66	–	00:22:03	–	–
Guided 1	00:22:12	58	–	58	–	00:22:12	–	–
Guided 1	00:20:21	67	67	–	–	00:20:21	–	–
Narrative	00:27:55	67	67	–	00:27:55	–	–	–
Narrative	0:48:00	26	–	26	0:48:00	–	–	–
Guided 2	00:23:06	70	70	–	–	–	00:23:06	–
Guided 2	00:13:59	47	47	–	–	–	00:13:59	–
Guided 2	00:25:40	72	–	72	–	–	00:25:40	–
Guided 2	01:43:11	25	25	–	–	–	01:43:11	–
Guided 2	00:38:24	42	–	42	–	–	00:38:24	–
*Expert physio*	*00:16:14*	*n/a*	*n/a*	*–*	*–*	*–*	*–*	*00:16:14*
*Expert surgeon*	*00:56:10*	*n/a*	*–*	*n/a*	*–*	*–*	*–*	*00:56:10*
*Expert support group*	*00:56:10*	*n/a*	*–*	*n/a*	*–*	*–*	*–*	*00:56:10*
**Total**	**09:47:41**	**–**	**8**	**8**	**01:15:55**	**02:58:52**	**03:24:20**	**02:08:34**
***Mean***	00:36:44	50.8	50.1	51.7	00:37:58	00:29:49	00:40:52	00:42:51
***Median***	–	50	50	52	00:37:58	00:22:08	00:25:40	00:56:10
***min***	00:13:59	25	25	26	00:27:55	00:14:10	00:13:59	00:16:14
***Max***	01:43:11	72	70	72	00:48:00	00:53:07	01:43:11	00:56:10

**Table 2:** Diagnoses and treatments of the respondent. Expert interviews are not listed here (interview numbers 14–16)

IP	Interview form	Sex	Age (years)	Diagnosis	Treatment/revisions
1	Guided 1	F	25	Multiple myeloma left clavicle, pathologic fracture	Osteosynthesis left clavicle
2	Guided 1	F	50	Epithelioid sarcoma left upper extremity. Fistulating inflammation in the area of the acromioclavicular joint	Humerus disarticulation, wound revision including fistula excision and debridement
3	Guided 1	M	46	Myxofibrosarcoma of the left biceps brachii	Removal of left biceps brachii
4	Guided 1	M	66	Malignant giant cell tumor, Mechanical complication (extensive bushing wear) of a HMRS tumor endoprosthesis right dist femur	Resection and Implantation of HMRS tumor endoprosthesis, Two-step prosthetic exchange
				Low grade prosthetic infection	
5	Guided 1	M	58	? Malignant neoplasm: connective tissue and others soft tissue, unspecified	Resection of malignant bone and soft tissue tumors on the lower extremity
					Soft tissue tumor resection of the lower leg on the left
6	Guided 1	F	67	Synovial sarcoma inguinal, lymphatic fistula left groin	Soft tissue mass resection, tissue replacement by pedicled myocutaneous flap plasty, chemotherapy and radiation
7	Narrative	F	67	Osteosarcoma right prox. tibia chronically recurrent fistulant prosthesis infection	Resection and Implantation of KMFTR total knee endoprosthesis, multiple revisions
8	Narrative	M	26	Synovial sarcoma right knee joint	Implantation of a tumor endoprosthesis/resection of the knee joint
					Tissue replacement or reconstruction with a free flap
					Resection of malignant bone and soft tissue tumors on the lower extremity
9	Guided 2	F	70	Exoskeletal osteosarcoma of the left dorsal thigh, sequela of femur fracture	Osteosynthesis on the left thigh shaft Resection of malignant bone and soft tissue tumors on the lower extremity
10	Guided 2	F	47	Synovial sarcoma dorsal right knee	Transfemoral amputation right side
					Adenectomy of lymph node right groin
11	Guided 2	M	72	Pleomorphic sarcoma soft tissue resection right lateral vastus muscle	Open biopsy of tumor formation in the ventral distal thigh. Tumor resection lower extremity right
12	Guided 2	F	25	Ewing sarcoma right distal femur	GMRS Tumor endoprosthesis right distal femur
13	Guided 2	M	42	Osteosarcoma left distal femur	Mutars® (implant, implantcast GmbH, Buxtehude, Germany) total femoral replacement endoprosthesis status postreimplantation

*HMRS* Howmedica Modular Resection System, *KMFTR* (Kotz-Modular-Femur-Tibia-Rekonstruktionssystem) KMFTR (implant), *GMRS* Global Modular Replacement System

### Theoretical model

The developed theoretical model shows the main findings: the focus is on the affected person, the patient, who needs a supportive environment to achieve a sense of normality related to the level of movement and individuality of the affected person. The theoretical model and the extracted codes merge to form the core category: the affected person/patient. In order to develop a sense of normality and avoid the impression of incapability or uselessness, a well-functioning (and adapted) social surrounding is a basic requirement, namely the partner/relatives and the GP (Fig. 4). In the following paragraphs the model is explained in detail, square brackets and three dots are omissions for a non-relevant part of the text; the interviewee is marked with IP (interviewee/participant) and the number at the end of the quote.

#### Normality

It is important to find a sense of normality for those affected: This network consists of the following five elements: (1) daily routine, (2) leisure, (3) mobility, (4) family and (5) friends. The networks overlap and show the importance of these elements and what is needed to find in a routine or sense of normality (Fig. 1). This means, among other things, to be able to move to get dressed.

“[…] it’s nothing that you have to go through in everyday life as a couple […] and so far he’s been doing it super great […] after the major operation, he had to put on and take off my socks, put on and take off my underpants […].” IP 6

This quotation comes from a younger respondent, who was not married at the time, which was a very difficult situation for her and contradicted her sense of normality. Normality was also mapped with:

“So, this is what is most important to me and what also helps me. So that really nothing has changed […] yes, with going out and going to the cinema and no idea […].” IP 2

Before getting mobile from point A to B, the physical movement must be intact again. Normality is a part of movement and interacts with mobility.

#### Movement

Movement is the generic term summarizing several elements that explain what is necessary for the individual to move or to be active (Fig. 2). These six elements (1) daily routine, (2) normality, (3) leisure, (4) mobility, (5) sense of self and (6) sport are interrelated. Movement is important for daily routine, for one interviewee, it was riding a bicycle, for another it was cleaning windows or to swimming:

“Look, get mobile as soon as possible. Work, cycle, hike as quickly as possible.” IP 4

“[…] and then yes I like to go swimming.” IP 7

“I asked the doctor: Yes, I can clean windows because of the hand? (laughing).” IP 1

Independence is the most important factor that those affected can achieve. Therefore, movement is necessary and important to get a sense of normality.

#### Individuality

The third network describes dependency and that the individuality and personality of the person affected are very important points for, e.g. compliance (Fig. 3). Thus, persons with long-term diseases need holistic treatment and collaboration of the health professionals and related disciplines. The following quotation is from a very positive young man, who was treated by an amputation of the left upper extremity:

“I’ve always been a very independent person. So, it is a bit gnawing that some things are more difficult or sometimes not possible at all. However, it doesn’t bother me excessively now (pause).” IP 2

Another young female, wearing a tumor prosthesis on her right lower extremity with a different mindset (I = interviewer, P = participant):

I: “You said you did play a lot of sport before your treatment and now you don’t play at all. Not even riding a bike or something?”

P: “No, I’m afraid of falling. According to the surgeon, I should come to the hospital and have an x‑ray. Only then, I would be there every week and that wouldn’t interest me either.” IP 12

#### Relatives and partners

Close relations are crucial and provide the greatest support from the husband/wife to the beloved pet. As a rule, the closest relatives e.g. mother or children are the most important people supporting the affected person. In addition, the inner circle includes the closest friends. An interesting finding in this sampling was the “partner” issue was mixed, some had a good relationship and received support, others did not provide deeper insight. Quotations justifying this category:

Close contact with family, who live 15 minutes away by car, ensures regular support. IP 4

Another patient said: “So, my close circle of friends, they handle me as always.” IP 2

Furthermore, the beloved dog has a crucial role:

“My dog even visited me in front of the hospital.” IP 12

Not everyone is strong enough when a friend is suffering from a malignant disease:

“And she only visited me once in the hospital during the entire illness but told me that she couldn’t handle it psychologically. Because she has been to hospitals so often because of her family that she can’t make it and she apologized 100 times anyway, that’s OK for me.” IP 2

#### General practitioner (GP)

The GP is important for primary health care and for supporting the sick. The poor communication between hospital and GP is problematic. The family doctor is considered the first point for health issues. The GP is important for helping patients plan further treatment or therapies, such as blood taking. The following quotations highlights the importance of collaboration between GP and specialists and suggest that it should be intensified.

“[…] so that belongs in a specialized center. The family doctor is a support for the patient for other organizational areas. Further prescription of pain medication. Initiate rehabilitation if we fail to do so. […].”

“Many general practitioners call and want the surgery report. This is of course welcomed.” IP Surgeon

“Yes, it should be promoted more intensively; improved communication by the specialists is needed.” IP SH

Patients need the support from their family and life partners to experience a sense of normality and avoid the impression of incapability or uselessness. It is noted that individuality and personality are necessary for a sense of physical health during and after treatment. However, all factors seem to work together in the patients’ perception to create the impression of normal daily activity and routine, as before the disease. For those affected, it was important to be independent by regaining mobility and function as quickly as possible. For one of the affected persons, it was riding a bike, for another it was cleaning the windows. These activities gave them a sense of normality, which was experienced as important to the recovery process. This also includes support from family and friends. The networks of movement and personality interact with each other in favor of the affected person’s self-esteem. How one “connects” with other persons is also important for the individuality. Therefore, self-perception and body image are important factors in feeling healthy and being part of society.

In our study, the involvement of the closest relative or significant other person is crucial to achieve this result. In addition, it can be stated that the GP is the hub for most of the patient’s medical needs and can assist the patient in various matters, such as coordinating administrative matters:

“The hospital does not manage to have the MR and CT on the same day, no, there are 2 weeks in between. That means what can you do again then? Another blood sample.” IP 12

## Discussion

The main aim of this study was to investigate how patients return to their daily routine after tumor surgery and/or treatment and what has changed for them. This resulted in the person concerned being listed as the main category and the main coping strategy was an interacting system of movement, normality and individuality. Patients diagnosed with musculoskeletal malignancies face many changes in their lives from the time of diagnosis, including treatment: radiotherapy, chemotherapy, surgery and so forth. People diagnosed with primary or secondary bone cancer or other malignant neoplasm are more likely to be long-term survivors. Patients with musculoskeletal tumors who are longer term survivors rely on a more holistic approach that addresses other important aspects of their lives and individuality in addition to medical support.

They developed coping mechanisms to find a new routine, to find a new way of normality [26]. Participants described what changed and that there were obstacles in their daily lives, some negative aspects, and other problems that had to be overcome. These problems correlated with impaired physical function and changes in the treated limb [27]. In the present qualitative study, all patients were primarily concerned with maintaining their daily routines and activities. In addition, they were anxious to avoid too much support from their social environment. It was of great importance for them to find coping strategies to move freely and regain a sense of normality. For patient, numerous coping strategies and changes in daily habits are necessary to get used to a new routine [2]. To find the new routine and normality, both movement and social support are essential. This is consistent with recent qualitative studies on all-cancer studies [3, 6, 28, 29].

The term movement encompasses not only the level of physical activity required for certain sports or recreational activities, but also movement sequences necessary to accomplish everyday routines, such as cooking, cleaning the home, doing gainful employment and household chores on one’s own [6]. Some degree of mobility is necessary for independence. All participants want to achieve or maintain a certain level of independence; moreover, this is important for the recovery process.

The self-perception of the new body image interacts with this process and is individual for each person concerned. Healthy, athletic, active people are the socially shaped ideal, propagated in mass and social media [30–32]. Personality and identity of cancer survivors are interdisciplinary research topics and should always be kept in mind when communicating research questions [33–35]. In this study, two participants were extremely conflicted in their opinions (IP 2 and 12). Both are young and have been diagnosed with sarcoma. One is very positive and gives the feeling of having accepted his diagnosis. The other one is angry at her surroundings, at everything and the world; she says itis unfair that she has become ill.

Humans need social networks to cope with everyday life. This is even more important in the case of an illness. Social support was an important issue in our patients [8–11] The information obtained here will help us to deal more appropriately with certain aspects such as the involvement of relatives, etc. Relationships are a delicate issue: on the one hand, they should provide physical and emotional support, and on the other hand, they should provide enough independence. In addition, there are some negative aspects in relationships mentioned in cancer patients. However, many of the people in the social environment have a distanced attitude towards the ill person. Therefore, the existing relationships are of crucial importance [27].

Many of our findings on social support and information of the affected person are related to the GP. This is consistent with Hoffmann et al., who described that individuals with a chronic condition visit the GP more often than persons without chronic conditions [36]. Patients with longstanding illness (including oncological diseases) rely on the GP for prescribing pain medication, organizing treatment appointments, or general health-related recommendations.

For oncology patients, the GP or support groups can be important as gatekeepers and stakeholders [37]. Medical language, which includes specific abbreviations, a rapid pace of speech, and different phrases, leaves the patient with many questions that can be answered by the GP [38–40]. Discussion with the GP is necessary to understand and process the information as the informed patient develops better coping strategies and is aware of possible treatment options [41–43]. A network should be established that includes the GP, the patient, and the caregiver from family or friends. Several important needs should be solved in this network. Participants criticized poorly organized visits to the hospital, which do not seem to be well organized, e.g., multiple appointments on different days.

Hoffmann et al. pointed out that Austria has one of the highest utilization rates of healthcare services; this could be due to the lack of a clear distinction between primary and secondary health care [44].

Our findings support the statement of the current health care reform to shift inpatient treatment to the outpatient setting, with a focus on improving primary care [45]. In Scandinavian countries, e.g. Sweden and Norway, this is also a regulatory gatekeeping mechanism. Furthermore, Austria has one of the highest numbers of hospital beds in Europe, while on the other hand, the training of GP and family physicians lags far behind European and international standards [44, 46]. This could also be a reason for the poor communication between the hospital and primary care; patients are in the middle and have to communicate their needs and requirements, often several times. This takes a lot of time for long-term patients and is frustrating; it is even more difficult for the old.

This study was conducted to find the right questions, not the right answers. While it is a process between all parties involved and, according to our findings, it is important to listen carefully to what the patient is saying, health professionals should simplify their language and adapt their recommendations to the personality of the individual. Consequently, these questions will provide us with more holistic information to further improve treatment, patient support, and communication between healthcare providers and patients. A qualitative approach is used to discover real life rather to present numerical representativity [47]. From the outset, the scope of the results was limited to the sample described, and conclusions can only be drawn from this empirical, specified domain.

However, some limitations should be noted. First, because of the infrequency of diagnosis of musculoskeletal malignancies, a broad and open recruitment plan was established, which could lead to selection bias. Therefore, homogeneity of diagnosis or cancer staging cannot be demonstrated. There is also no previous data from Austria in this area, which is why no comparison could be made. Second, the first author conducted the analysis mainly with assistance of different peer groups. It was not consistent for each interview to be coded by more than one author, which increased the study time. In qualitative studies, the work and role of the authors must be reflected and described. To this end, it is worth noting that authors are familiar with the field but inexperience is recommended in classical grounded theory. Charmaz states that neither data nor theories are discovered, they are developed [18]. Furthermore, the results are meaningful for the study population presented.

Based on these results, questionnaires can be developed and evaluated with a larger sample in order to secure and further develop these results. Likewise, collaboration with other centers should be considered to increase coverage.

In conclusion, specialized trained health professionals should support the patient’s social environment, especially the communication channels to the GP, relatives and partners. Patients are overwhelmed with administrative matters or treatment appointments; they want to save their free time and energy for their recovery.

## Supplementary Information

Supplement 1 Opening question for the narrative interviews

Supplement 2 Guideline—Experts

Supplement 3 Interview Guideline 1

Supplement 4 Interview Guideline 2
