# Supplementary material for: Patients’ perception of changes and consequences after tumor resection: A qualitative study in Austrian patients with musculoskeletal malignancies
Source: Wien Klin Wochenschr. 2023 Jan 3;135(11-12):301–10. doi: 10.1007/s00508-022-02136-6 (PMC10287576; doi:10.1007/s00508-022-02136-6)
Supplement: Supplementary file 1 — Supplement 1 Opening question for the narrative interviews [file 508_2022_2136_MOESM1_ESM.docx]

Supplement 1

Opening question for the narrative interviews

Due to your illness, you have been through a lot of treatments, operations and analysis. Could you please explain your experience and how this process influenced your attitude towards life?
