# Supplementary material for: Patients’ perception of changes and consequences after tumor resection: A qualitative study in Austrian patients with musculoskeletal malignancies
Source: Wien Klin Wochenschr. 2023 Jan 3;135(11-12):301–10. doi: 10.1007/s00508-022-02136-6 (PMC10287576; doi:10.1007/s00508-022-02136-6)
Supplement: Supplementary file 2 — Supplement 2 Guideline—Experts [file 508_2022_2136_MOESM2_ESM.docx]

Supplement 2

Guideline - Experts

Qu 1

What are your experiences and recommendations regarding patient mobility after an oncological surgery?

Qu 2

What are your experiences and recommendations with regard to mobility if the patient has had chemotherapy and / or radiation?

Qu 3

How do you motivate your patients?

Qu 4

To what extent are relatives / life partners included in the treatment / discussion of the findings?

Qu 5

What are your experiences and recommendations in working with your family doctor? Before and after treatment?

Qu 6

Do you talk about personal matters with the patient?

Qu 7

How long have you been working in your job?
