# Supplementary material for: Patients’ perception of changes and consequences after tumor resection: A qualitative study in Austrian patients with musculoskeletal malignancies
Source: Wien Klin Wochenschr. 2023 Jan 3;135(11-12):301–10. doi: 10.1007/s00508-022-02136-6 (PMC10287576; doi:10.1007/s00508-022-02136-6)
Supplement: Supplementary file 3 — Supplement 3 Interview Guideline 1 [file 508_2022_2136_MOESM3_ESM.docx]

Supplement 3

Interview Guideline 1

**I. Consultation history**

1. Please tell me about your illness and your treatment.

2. How did you get your diagnosis?

3. Are or were there people which recommended your current physician?

**II. General perception**

1. How present is your illness in your daily life?

2. Are you well informed about your illness?

3. How is your understanding of your illness after the operation?

4. What feelings did you have during your hospital stay? Fear / pain / hope / depression?

5. In what situations did you have these feelings?

6. Did the doctor talk about something other than your illness? For example, job - incapacity for work / sick leave.

**III Socio-demographic**

1. How old are you?

2. Are you currently employed?

3. If yes, full or part time?

4. If not, retirement or sick leave?

5. What occupation do / did you have?

Would you like to tell me something else?

Thank you for your cooperation!
