# Supplementary material for: Patients’ perception of changes and consequences after tumor resection: A qualitative study in Austrian patients with musculoskeletal malignancies
Source: Wien Klin Wochenschr. 2023 Jan 3;135(11-12):301–10. doi: 10.1007/s00508-022-02136-6 (PMC10287576; doi:10.1007/s00508-022-02136-6)
Supplement: Supplementary file 4 — Supplement 4 Interview Guideline 2 [file 508_2022_2136_MOESM4_ESM.docx]

Supplement 4

Interview Guideline 2

**I. Consultation history**

1. Please tell me about your illness and your treatment.
2. Are or were there people which recommended your current physician?
3. Which therapies did you have and how did you find out about it?
4. Would you like to tell me something about your illness?

**II. General perception**

1. How are you today?
2. Tell me about your hobbies / what do you do in your free time?
3. How important is sport to you? Did this change?
4. Would you tell me about your family / home?
5. Where are you going on your next vacation?
6. How do you explain your illness to someone else?
7. What thoughts do you have recently?

**III Socio-demographic**

1. How old are you?
2. Are you currently employed?
3. If yes, full or part time.
4. If not, retirement or sick leave?
5. What occupation do / did you have?

Would you like to tell me something else?

Thank you for your cooperation!
